# Whole-genome characterization of halotolerant *Enterobacter roggenkampii* OSNO4 and its potential for climate-resilient agriculture

**DOI:** 10.1371/journal.pone.0351555

**Published:** 2026-06-18

**Authors:** Sanjoy Kumar Mukharjee, Md. Faruk Hasan, Biswanath Sikdar

**Affiliations:** 1 Department of Microbiology, University of Rajshahi, Rajshahi, Bangladesh; 2 Department of Microbiology, Noakhali Science and Technology University, Noakhali, Bangladesh; 3 Department of Genetic Engineering and Biotechnology, University of Rajshahi, Rajshahi, Bangladesh; Benemérita Universidad Autónoma de Puebla: Benemerita Universidad Autonoma de Puebla, MEXICO

## Abstract

Plant growth-promoting rhizobacteria (PGPR) represent an eco-friendly strategy to improve crop yield under abiotic stress conditions. This study aimed to perform a comprehensive genomic and functional profiling of a halotolerant rhizobacterium to evaluate its multi-trait plant growth-promoting (PGP) potential and its precise contribution to mitigating salinity stress for climate-resilient agriculture. In the present study, 24 (8.5%) rhizobacterial isolates showed phosphate-solubilizing activity out of 283 isolated bacteria from rice rhizosphere. From these, strain OSNO4 was selected for detailed evaluation. The isolate demonstrated phosphate solubilization (solubilization index: 1.22) and potassium solubilization, indole-3-acetic acid (IAA) production (38.34 µg/ml), nitrogen fixation, siderophore and ammonia production, protease activity, and biofilm formation *in vitro*. Additionally, it showed tolerance to salinity (10% NaCl), drought (20% PEG 6000), temperature (45 °C), and a broad pH range. Strain OSNO4 further suppressed the growth of phytopathogenic fungi *Fusarium concentricum* by 54.23%. Whole-genome sequencing analysis identified the strain as *Enterobacter roggenkampii* (ANI 98.2%, dDDH 85.7%), with a 4.67 Mb genome harboring total 4,546 predicted genes and diverse functional subsystems. Comprehensive genomic study further revealed the presence of genes linked to nutrient mobilization, phytohormone biosynthesis, abiotic stress tolerance, and antifungal activity. Six biosynthetic gene clusters, including siderophore-related and putatively novel clusters were also identified. Pan-genome analysis revealed an open genome structure with high flexibility and genetic variability. Under salinity stress, OSNO4 inoculation significantly improved rice seed germination (52.22% to 85.56% at 100 mM NaCl; 42.22% to 72.22% at 150 mM NaCl), root and shoot development, and biomass accumulation compared to uninoculated controls. The strain also showed broad antibiotic susceptibility and non-hemolytic phenotype, suggesting its biosafe nature. In summary, these data demonstrate that *E. roggenkampii* OSNO4 possesses a robust repertoire of genomic determinants and functional capabilities, establishing it as a highly potent bioinoculant for deployment in climate-resilient and sustainable agroecosystems.

## Introduction

Soil salinity is recognized as one of the most critical abiotic stresses restricting global agricultural productivity and food security, particularly within rice-cultivating regions [1]. Rice (*Oryza sativa* L.), a staple food crop for more than half of the world’s population, exhibits extreme sensitivity to salinity-induced stress [2]. Excessive salt accumulation impairs nutrient acquisition, osmotic balance, photosynthesis, and cellular homeostasis, ultimately resulting in severe yield losses [3]. Consequently, the prospecting of halotolerant plant growth-promoting rhizobacteria (PGPR) capable of improving rice growth under saline conditions has become an important research priority for sustainable agriculture and climate adaptation strategies [4]. Several halotolerant rhizobacteria belonging to genera such as *Bacillus*, *Pseudomonas*, and *Enterobacter* have demonstrated remarkable efficacy in enhancing plant growth and stress resilience under saline environments [5–10]. However, despite increasing reports of salt-tolerant PGPR, numerous investigations remain confined to phenotypic characterization, while comprehensive genome-guided investigations linking stress adaptation, biosynthetic potential, ecological fitness, and biosafety profiles remain relatively sparse.

Among numerous PGPR, species of the genus *Enterobacter* have attracted significant attention because of their remarkable metabolic flexibility and ecological adaptability [11,12]. A diverse array of *Enterobacter* strains isolated from agricultural soils and plant rhizospheres have been reported to possess multiple plant growth-promoting (PGP) features, including phosphate solubilization, nitrogen metabolism, and indole-3-acetic acid (IAA) production [13–16]. Several *Enterobacter* species, including *E. cloacae*, *E. ludwigii*, and *E. hormaechei*, have been the subject of detailed genomic investigations that unveiled robust repertoires of biosynthetic gene clusters, secondary metabolite pathways, and stress-responsive regulons [17–20]. These genomic insights have been pivotal in transitioning PGPR research beyond phenotypic characterization toward a mechanistic understanding of plant-microbe dialogue at the molecular level [21].

While detailed genomic characterization of other plant-beneficial *Enterobacter* strains is available, the whole-genome exploration of plant-associated *Enterobacter roggenkampii* remains only limited [15,22]. This is a notable gap, as *E. roggenkampii* has been isolated from diverse ecological niches, including rhizosphere, implying the possession of ecophysiological traits relevant to plant association [15,22–24]. The scarcity of genome-scale data for this species dictates that its entire complement of PGP determinants, stress tolerance mechanisms, and biosynthetic potential remains largely uncharacterized [25,26]. Furthermore, because *E. roggenkampii* exhibits close phylogenetic affinity to clinically relevant members of the *Enterobacter cloacae* complex, a rigorous biosafety assessment, encompassing antibiotic resistance gene profiles, virulence factor repertoires, and mobile genetic element content is essential before any agricultural deployment can be considered [12,27]. Recent advances in high-throughput sequencing and bioinformatics have rendered whole-genome sequencing an indispensable tool for the comprehensive evaluation of PGPR strains [28]. Functional annotation pipelines, secondary metabolite prediction platforms such as antiSMASH, and virulence/resistance databases (e.g., VFDB, CARD) now facilitate a holistic, evidence-based assessment of both the beneficial and potentially hazardous attributes of candidate strains in a single analytical framework [29–31]. In particular, pan-genomic analysis has emerged as a powerful approach to delineate the core functional genome shared across a species from accessory elements that confer strain-specific ecological adaptations [32]. Integrating these tools offers an avenue to rigorously characterize PGPR strains in a manner that simultaneously addresses agronomic promise and regulatory biosafety concerns [33].

In the present research, a promising halotolerant rhizobacterial strain, *E. roggenkampii* OSNO4, was isolated from the rhizosphere of rice (*Oryza sativa* L.) and characterized using an integrated phenotypic and genomic approach. Whole-genome sequencing, functional annotation, and comparative analyses were conducted to elucidate PGP attributes, stress adaptation mechanisms, and biosynthetic potential. Additionally, pan-genome analysis and evaluation of antibiotic resistance and virulence-associated genes were performed to assess ecological positioning and safety profile. The study thus addresses a critical knowledge gap regarding *E. roggenkampii* and provides a genome-guided blueprint for evaluating its suitability as a safe and effective bioinoculant for salinity-stressed rice cultivation.

## Materials and methods

### Ethical statement

No specific permits or permissions were required for the field sampling in this study, as the rhizobacterial strains were isolated from common agricultural rice rhizosphere soil that is not privately owned or protected. Additionally, the fieldwork did not involve any endangered or protected species.

### Soil sample collection, bacterial isolation and preliminary screening

A total of twenty-nine rhizosphere soil samples were collected from healthy rice plants cultivated across six districts (Rajshahi, Natore, Rangpur, Khulna, Noakhali, and Lakshmipur) in Bangladesh. The plants were uprooted, and root-adhering soil was aseptically collected into sterile polybags. The samples were then transported to the laboratory under cold conditions, and soil pH was measured immediately. Isolation of rhizobacteria was carried out using the serial dilution spread plate method on nutrient agar (NA) media.

Morphologically distinct colonies were selected, and following repeated streaking on NA media, isolates were purified. The purified isolates were initially screened for their phosphate-solubilizing potential on Pikovskaya’s (PVK) agar medium using the spot inoculation method [34]. Following incubation at 28 ± 2 °C for 5–7 days, the formation of clear halo zones surrounding the bacterial colonies was considered indicative of positive phosphate solubilization. These isolates were subsequently preserved for further characterization and analysis.

### *In vitro* plant growth-promotion, abiotic stress tolerance and biocontrol traits

The phosphate-solubilizing rhizobacterial isolates were next evaluated for various plant growth-promoting traits to assess their biofertilization capabilities. Phosphate [34] and potassium solubilization [35,36] were evaluated by inoculating the isolates onto Pikovskaya’s and modified Aleksandrov agar media, respectively, where the formation of halo zone around the bacterial colony indicated positive mineral mobilization. Indole-3-acetic acid (IAA) production was determined calorimetrically [37] by growing the isolates in Luria-Bertani (LB) broth supplemented with 0.1% L-tryptophan; the development of a pink-to-red color upon the addition of Salkowski’s reagent confirmed IAA synthesis. Nitrogen fixation capacity was initially screened using nitrogen-free Jensen agar media, supplemented with bromothymol blue, observing for intensified blue color surrounding the colonies [38]. Siderophore production was assessed using the Chrome Azurol S (CAS) agar assay, where a color change from blue to orange/yellow around the colony demonstrated iron-chelation activity [39]. Protease activity was detected by clear zone formation on skim milk agar plates [40]. Ammonia production was identified by the development of a brown/yellow color upon adding Nessler’s reagent to peptone broth cultures [41]. Hydrogen cyanide (HCN) production was observed via the color change of filter paper soaked in picric acid and sodium carbonate from yellow to light brown or reddish-brown [42]. Biofilm formation was determined using the conventional test tube method with crystal violet staining, observing purple rings along the inner surfaces of the test tubes for positive isolates [43].

Most potent PGPR were further tested in LB broth under shaking conditions for different abiotic stress tolerances such as drought (by supplementing the medium with PEG 6000, 5–25%) [44], salinity (by adjusting NaCl concentrations from 2.5–10%) [45], temperature (incubating at 30–50 °C) [45], and pH (adjusting broth pH from 4–12) [45] as previous reports. Bacterial growth under these stress regimes was monitored spectrophotometrically, and optical density (OD) values of ≥ 0.1 at 600nm were considered positive test results.

Biocontrol activity of the isolates against *Fusarium fujikuroi* (MT856372) and *Fusarium concentricum* (MT856371) was determined employing the dual culture antagonistic method on Potato Dextrose Agar (PDA) plates [43], where the radial growth of the fungal mycelium towards the bacterial colony was measured after incubation. The inhibition percentage was calculated relative to the control plate, and a value ≥ 50% was considered potential biocontrol ability of the isolates.

### Preliminary identification of isolate OSNO4

Based on the overall performance in PGP traits, abiotic stress tolerance, and biocontrol assays, isolate OSNO4 was selected for detailed genomic and functional characterization. Preliminary identification of the isolate was carried out through morphological and biochemical analyses, along with *16S rRNA* gene sequencing. Morphological characterization included assessment of colony size, shape, margin, elevation, texture, opacity, color, and odor. Biochemical tests comprised Gram staining, catalase and oxidase activities, motility, triple sugar iron (TSI) test, indole production, Methyl Red-Voges Proskauer (MR-VP) tests, citrate utilization, urease activity, and nitrate reduction [41].

Genomic DNA was extracted using the Monarch^®^ Genomic DNA Purification Kit (New England Biolabs, Inc., USA) following the manufacturer’s instructions. The *16S rRNA* gene was amplified using universal primer set (27F and 1492R), followed by sequencing of the PCR products. The generated sequences were edited using BioEdit [46], and sequence homology was obtained through Basic Local Alignment Search Tool (BLAST) [47]. Multiple sequence alignment was performed using ClustalW [48], and phylogenetic relationships were inferred using MEGA software [49].

### Whole-genome sequencing, genome assembly and functional annotation

For whole-genome sequencing of strain OSNO4, Illumina NextSeq 2000 platform was employed at the Genome Centre (iGC) of the International Centre for Diarrhoeal Disease Research, Bangladesh (Dhaka, Bangladesh). Raw sequence reads were initially assessed for quality using FastQC v0.12.1 [50], followed by quality filtering and trimming with FastP v1.0.1 [51]. De novo genome assembly was conducted using SPAdes v3.15.5 [52], and the quality of the assembled genome was evaluated using QUAST v5.3.0 [53]. Circular genome map was constructed using the Proksee server [54]. The corresponding genome assembly has been archived in GenBank under the accession numbers PRJNA1344764 (BioProject) and SAMN53636785 (BioSample). The draft genome was annotated using Prokka v1.14.6 [55] for downstream analysis. Functional categorization into biological subsystems were performed using the Rapid Annotation using Subsystem Technology (RAST) web server [56].

### Phylogenetic analysis

The whole-genome sequence of strain OSNO4 was subjected to phylogenetic analysis using the Type Strain Genome Server (TYGS) [57] to confirm its taxonomic position. Phylogenetic reconstruction was carried out with FastME v2.1.6.1 [58], employing Genome BLAST Distance Phylogeny (GBDP) metrics calculated from whole-genome data. Branch lengths were assessed using the GBDP distance formula *d*_*5*_, and the tree was rooted at the midpoint [59]. Genome-level relatedness between the strains was determined through digital DNA-DNA hybridization (dDDH) using the Genome-to-Genome Distance Calculator (GGDC) tool [60] implemented within TYGS. In addition, average nucleotide identity (ANI) was calculated via the JSpeciesWS platform [61]. A threshold value of 70% dDDH [62] and ≥ 95–96% ANI [63,64] was considered indicative of species-level delineation, following accepted taxonomic guidelines.

### Genome mining for PGP traits and secondary metabolites

To predict plant growth-promoting traits (PGPTs), the genome of OSNO4 was analyzed using the PGPT-Pred tool of PLant-associated BActeria web resource (PLaBAse) [65]. The module detect plant associated genetic determinants using a combined BLASTP and HMMER methodology. Secondary metabolite biosynthetic gene clusters (BGCs) were detected using antiSMASH v8.0.4 [29].

### Genes related to antimicrobial resistance and pathogenicity

The genome of OSNO4 was examined for biosafety-relevant genes using ABRicate v1.0.1 [66] on the Galaxy platform. Comprehensive Antibiotic Resistance Database (CARD) [67] and Virulence Factor Database (VFDB) [68] were employed for identifying antimicrobial resistance and virulence-associated genes respectively.

### Pan-genome analysis

Pan-genome analysis of strain OSNO4, together with 24 closely related *E. roggenkampii* genomes, was conducted using Roary v3.13.0 [69] on the Galaxy server. At first, high-quality genome sequences representing both environmental and clinical isolates of *E. roggenkampii* were retrieved from the NCBI database. All genomes were re-annotated using Prokka v1.14.6 [55] for uniform annotation, and the resulting GFF files were used as input for Roary with a BLASTp identity cutoff of 95% for gene clustering. The gene presence-absence matrix generated by Roary was subsequently used for comparative genomic analyses. Core genome-based phylogenetic relationships were inferred using RAxML [70] within the Galaxy platform, and the resulting phylogenetic tree was visualized using the Interactive Tree of Life (iTOL) tool [71].

### Effects of *E. roggenkampii* OSNO4 inoculation on rice seed germination under salt stress

*In vitro* germination of rice (*Oryza sativa* L., BRRI Dhan 28) seeds was tested following priming with OSNO4 (10⁸ CFU/ml) for 30 min; controls were treated with sterile water. Seeds were incubated on sterile, moistened filter paper at 28 ± 2 °C under a 12 h photoperiod and subjected to 50–200 mM NaCl. Germination rate was determined as radicle emerged within a few days [72,73].

For pot experiments, five-day-old seedlings were transplanted into double-autoclaved sandy loam soil (200 g per 7 × 10 cm pot). Seedlings were inoculated with OSNO4 (20 ml per pot) at 1 and 7 days after transplanting. Salinity (100 or 150 mM NaCl) was imposed every two days following the second inoculation. Pots were kept at 26 ± 2 °C under a 16/8 h light/dark cycle. After three weeks, root and shoot lengths, fresh biomass, and dry weight (after 48 h at 70 °C) were recorded [8].

### Antibiotic sensitivity and hemolysis test

Biosafety screening of OSNO4 included antibiotic susceptibility testing via disk diffusion method using piperacillin-tazobactam (100/10 μg), ceftazidime (30 μg), cefepime (30 μg), aztreonam (30 μg), gentamicin (10 μg), amikacin (30 μg), tobramycin (10 μg), ciprofloxacin (5 μg), imipenem (10 μg), meropenem (10 μg) [74]. Pathogenic potential of the isolate was assessed using hemolytic activity on 5% defibrinated sheep blood agar and after 24–48 h at 28 ± 2 °C the hemolysis was classified as α (green), β (clear), or γ (absent), with γ indicating non-hemolytic, safe strains [75].

### Statistical analysis

All experiments were replicated three times, and data were analyzed using SPSS v20.0. Mean comparisons among treatments were performed using Duncan’s multiple range test (DMRT) [76] at P ≤ 0.05.

## Results

### Isolation, *in vitro* plant-beneficial features and preliminary identification of strain OSNO4

**Fig 1:**
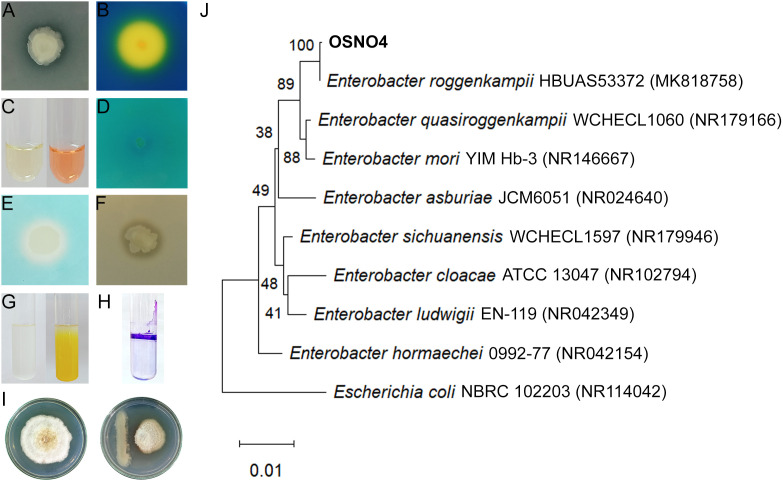
*In vitro* plant growth-promoting (PGP) traits of isolate OSNO4 and preliminary molecular identification. **(A)** Phosphate solubilization, **(B)** potassium solubilization, **(C)** indole-3-acetic acid production (control shown on the left), **(D)** nitrogen fixation, **(E)** siderophore production, **(F)** protease production, **(G)** ammonia production (control shown on the left), **(H)** biofilm formation, **(I)** antifungal activity against *Fusarium concentricum* (control shown on the left), and **(J)** phylogenetic analysis based on *16S rRNA* gene sequences, with OSNO4 highlighted in bold.

Out of 283 rhizobacterial isolates, 24 (8.5%) demonstrated phosphate-solubilizing activity (S1 Data) and were subsequently evaluated for *in vitro* plant growth-promoting (PGP) traits, from which 15 isolates were selected for further evaluation of abiotic stress tolerance and antifungal activity (S2 Data). Among these, isolate OSNO4 was chosen for comprehensive genomic and functional characterization due to its strong salt tolerance, antifungal potential, and well-balanced PGP profile. The isolate exhibited phosphate solubilization (PSI 1.22), potassium solubilization, indole-3-acetic acid production (38.34 µg/ml), nitrogen fixation, as well as siderophore, ammonia, and protease production, along with biofilm formation (Fig 1A–1H). It also showed tolerance to multiple abiotic stresses, including drought (20% PEG 6000), salinity (10% NaCl), elevated temperature (45 °C), and a wide pH range (4–10). Additionally, OSNO4 displayed significant antifungal activity against *Fusarium concentricum* (54.23% inhibition) (Fig 1I). Based on biochemical features (S3 Data) and *16S rRNA* gene sequence analysis (Fig 1J), isolate OSNO4 was preliminarily identified as *Enterobacter* sp., with the sequence deposited in GenBank under accession number PX498030.

### Genomic characterization and functional annotation of strain OSNO4

**Fig 2:**
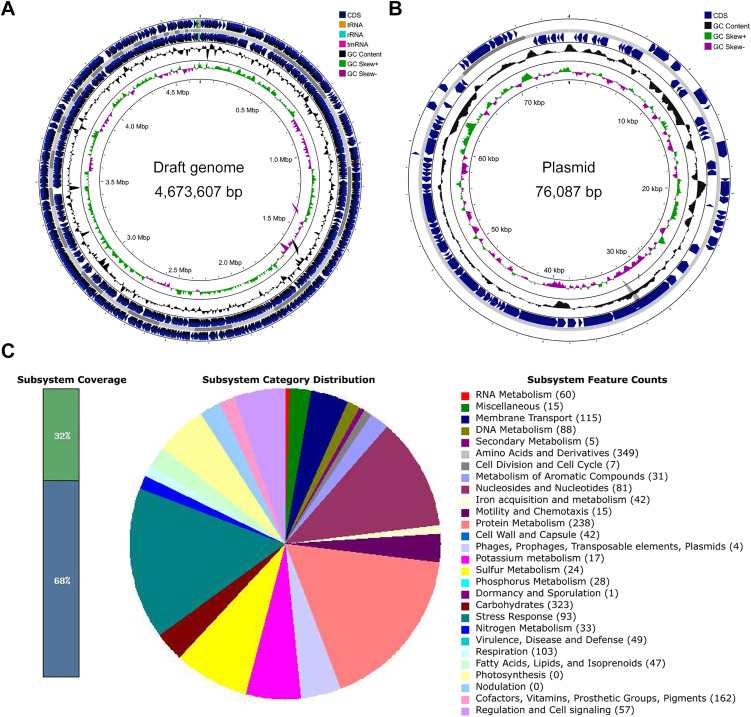
Circular genomic representation and functional annotation of strain OSNO4. **(A)** Circular map of the draft genome sequence, **(B)** circular map of the plasmid sequence, and **(C)** functional annotation of the genome based on RAST server analysis.

The whole-genome sequence of isolate OSNO4 was analyzed and deposited in the GenBank databases under the accession number JBSROR000000000. Circular maps of the draft genome and plasmid were generated using Proksee (Fig 2A, 2B). The genome comprises 4,673,607 bp with a GC content of 56.0% and harbors a single plasmid of 76,087 bp with a GC content of 54.4%. A total of 4,546 genes were predicted, including 4,372 protein-coding sequences, 52 pseudogenes, and 122 RNA genes (32 rRNAs, 84 tRNAs, and 6 ncRNAs), along with one plasmid. Assembly quality assessment using QUAST revealed 46 contigs, with an N50 value of 191,144 bp, an L50 of 8, an average genome coverage of 97.11 × , and an estimated completeness of 99.61% (Table 1).

**Table 1:** General genomic characteristics of strain OSNO4.

Characteristics		Strain OSNO4
Genome	Genome size (bp)	4,673,607
	GC content (%)	56.0
	Total genes	4,546
	Protein-coding genes (CDS)	4,372
	rRNAs (5S, 16S, 23S)	32 (12, 10, 10)
	tRNAs	84
	ncRNAs	6
	Pseudogenes	52
	Plasmid	1
	Plasmid size (bp)	76,087
	Plasmid GC content (%)	54.4
Assembly	Number of contigs	46
	N50 value (bp)	191,144
	L50 value	8
	Coverage	97.11×
	Completeness	99.61%

RAST subsystem annotation of the OSNO4 genome assigned 68% of the predicted coding sequences to functional subsystems, whereas 32% remained unclassified (Fig 2C). The functional distribution was largely dominated by fundamental metabolic categories, notably amino acids and derivatives (349 features), carbohydrates (323), protein metabolism (238), and cofactors, vitamins, prosthetic groups, and pigments (162), reflecting considerable metabolic versatility. Additionally, genes associated with environmental adaptation and cellular processes were well represented, including membrane transport (115), respiration (103), stress response (93), DNA metabolism (88), and regulation and cell signaling (57). Subsystems associated with iron acquisition and metabolism (42 features), nitrogen metabolism (33), metabolism of aromatic compounds (31), phosphorus metabolism (28) and motility and chemotaxis (15) further reflect rhizosphere competence and plant-associated functions of strain OSNO4.

### Phylogenetic analysis of strain OSNO4

**Fig 3:**
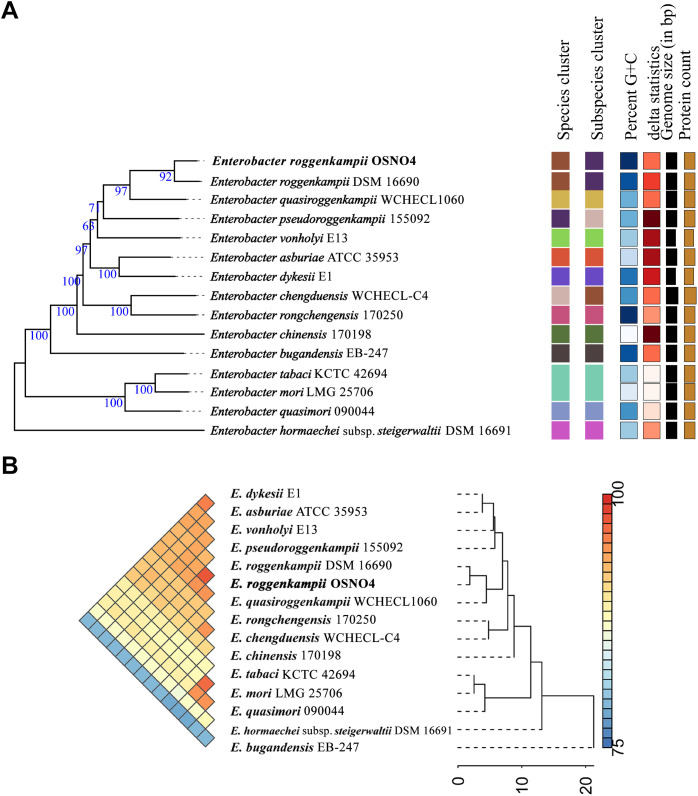
Phylogenomic analyses of *E. roggenkampii* OSNO4. **(A)** Genome BLAST Distance Phylogeny (GBDP) tree of OSNO4 generated using the TYGS platform. The values displayed above the branches represent GBDP pseudo-bootstrap support percentages exceeding 60% from 100 replicates, with an overall mean branch support of 93.3%. **(B)** Pairwise comparison of average nucleotide identity (ANI) values among OSNO4 and 14 other *Enterobacter* spp. genomes, generated using the Integrated Prokaryotic Genome and Pan-Genome Analysis (IPGA) web server.

Based on the whole-genome-based phylogenetic tree generated using TYGS, strain OSNO4 was found genetically similar to *E. roggenkampii*. (Fig 3A). Further, the dDDH (85.7%) and ANI (98.2%) values met the conspecificity requirements (dDDH > 70% and ANI ≥ 95%) to confirm the taxonomic classification of strain OSNO4 as *E. roggenkampii* (Fig 3B, S4 Data, S5 Data)

### Genome analysis for plant growth-promoting traits of *E. roggenkampii* OSNO4

**Fig 4:**
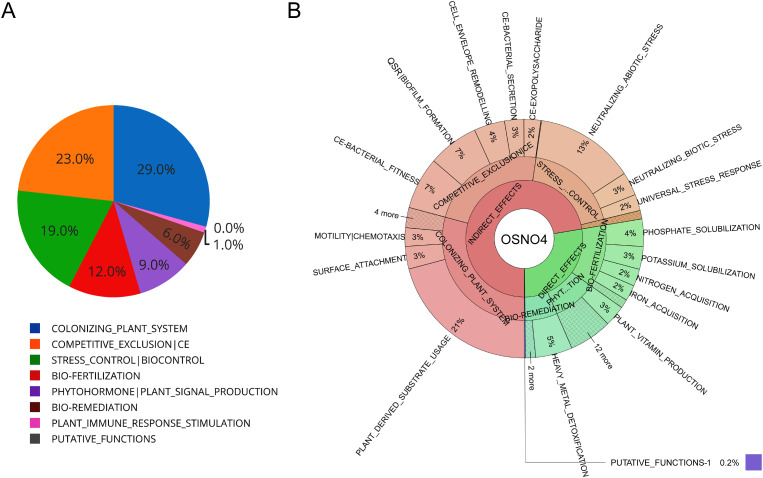
Genomic distribution and classification of plant growth-promoting traits (PGPTs) in *E. roggenkampii* OSNO4 genome. **(A)** Pie chart representing the genomic distribution of genes associated with PGPTs. **(B)** Krona plot illustrating the predominant PGPTs.

Genome analysis of OSNO4 using PGPT-Pred (PLaBAse) revealed diverse genetic determinants associated with both direct and indirect mechanisms of plant growth-promoting traits (PGPTs) (S6 Data). Overall, PGPT categories were dominated by colonizing plant system functions (29%), followed by competitive exclusion (23%), stress control/biocontrol (19%), biofertilization (12%), phytohormone production (9%), bio-remediation (6%), and plant immune response stimulation (1%) (Fig 4A). Within colonization-related functions, dominant categories include plant derived substrate usage (21%), surface attachment (3%) and motility or chemotaxis (3%). Genes related to competitive exclusion include biofilm formation (7%), bacterial fitness (7%), cell envelope remodeling (4%), bacterial secretion (3%) and exopolysaccharide production (2%). Stress tolerance-associated genes comprised neutralizing biotic and abiotic stresses, universal stress response, and induction of systemic resistance. Direct growth promotion traits included phosphate solubilization (4%), potassium solubilization (3%), nitrogen acquisition (2%) and iron acquisition (2%). Functions related to phytohormone production such as vitamin production (3%) and other associated plant growth-promoting mechanisms were also detected (Fig 4B).

The genome of *E. roggenkampii* OSNO4 possesses a comprehensive set of genes associated with nutrient solubilization and metabolism. Genes encoding high-affinity phosphate transport (*pstSCAB*) and regulatory components (*phoB, phoR, phoU*) were present, along with low-affinity transporter *pitA* and outer membrane porin *phoE*. Enzymes involved in organic phosphorus mineralization included alkaline phosphatases (*phoA, aphA*) and phytase-related genes (*phyA/B/C*). A complete phosphonate utilization cluster (*phnCDEFGHIJKLMNOP*) and genes involved in polyphosphate metabolism (*ppk, ppk2, ppx, ppa*) were also identified. Potassium solubilization and transport in OSNO4 is supported by a range of genes, including *kdpABCDE* operon for high-affinity potassium uptake, along with multiple low-affinity transport systems (*trkAH, trkD/kup, ktrAB,* and *kch*). Additionally, genes related to central carbon metabolism (*gltA, icd, mdh, sdhABCD, fumABC, aceEF, ackA-pta,* and *poxB*) were detected, including *pqq*-associated genes (*pqqF, pqqL, pqqI*).

The presence of tryptophan biosynthesis genes (*trpABCDEG, trpCF, trpS, trpR*), along with key IAA biosynthetic genes (*ipdC/ppdC*) and aminotransferase *aspC* supports the genetic basis of auxin (indole-3-acetic acid, IAA) biosynthesis pathways in *E. roggenkampii* OSNO4. Genes involved in aldehyde oxidation and conversion steps, such as *aldB, aldH/dhaS,* and *betB*-like homologs, were also present. Additional genes potentially contributing to auxin production and transport, including *iaaT/yedL/ysnE* and nitrilase-related genes (*nit*), were identified. Other associated metabolic genes (*patA1, puuE, lysN, mtr, solA, bsdC*) were also detected.

This genome harbors a broad repertoire of genes involved in nitrogen acquisition and metabolism. Key genes for ammonium uptake and assimilation (*amtB, glnA, gdhA, gltBD*), along with regulatory components (*glnB/K, glnD, glnE, ntrBC, rpoN*), were identified. Genes associated with nitrate and nitrite reduction (*narGHJI, narK, nasA, nirB, norVW*) were also present. The genome harbored a complete urease gene cluster (*ureABCDEFGJ*) with urea transporters (*urtABCDE*). Moreover, the presence of genes involved in nitrogen fixation (*nifJ, nifM, nifSU, hypABCDEF*) and nitrogen compound transport systems (*gltIJKL, glnHPQ*) suggests a broad capacity of nitrogen utilization.

In addition to nitrogen metabolism, the OSNO4 genome encodes a wide array of genes involved in iron acquisition, transport, and regulation. Identified components include siderophore biosynthesis and transport systems (*entABCDE, fepBCDEG, fepA, fhuBCD*), outer membrane receptors (*iroN, cirA*), ferrous iron uptake (*feoABC*) and iron storage genes (*bfr, ftnA*). Key regulatory elements, such as *fur* and two-component systems (*basRS, pmrAB*), were present. Furthermore, genes linked to heme biosynthesis (*hemABCD*) and multidrug/metal transport (*acrAB, tolC*) suggest effective iron homeostasis and environmental adaptability.

Osmotic and salinity stress tolerance by *E. roggenkampii* OSNO4 was supported by the presence of key osmoprotectant biosynthesis and transport systems, including glycine betaine (*betAB*) and transporters (*proVWX, proP*). The genome also encodes compatible solute metabolism involving trehalose (*otsAB*) and proline (*proABC*). Mechanosensitive channels (*mscL, mscS*) and ion regulation systems, including potassium uptake (*kdpABCDE*) and Na ⁺ /H⁺ antiporters (*nhaA, nhaB*), were detected. The isolate’s strong adaptive capacity to salinity stress was further evidenced by the presence of regulatory systems (*envZ/ompR, phoPQ*) and stress response genes (*osmY, dps*).

Genomic analysis of OSNO4 revealed multiple genes linked to antifungal activity and fungal stress control. These include genes involved in chitin-degrading and modifying enzymes (*chiA/chtA/chiI,* chitin deacetylase*, nagZ, chbG, bcsZ/wssD*), as well as those associated with carbohydrate metabolism (*tktA/B*, HEXA_B-like enzymes). Genes involved in polyamine biosynthesis (*speA*) and other metabolic activities (*ntdC, ybgC, lysS*) were present. Moreover, efflux and secretion systems, including *oprM/emhC*-type outer membrane proteins and associated transporters, further supports its stress adaptive potential.

Analysis of the *E. roggenkampii* OSNO4 plasmid revealed 94 coding sequences, including genes related to stability, adaptability, and potential dissemination of beneficial traits. Replication (*repA, repE*) and partitioning (*parM*) genes support stable maintenance, while stress response and DNA modification genes (*yhdJ, umuC,* and *umuD*) suggest enhanced environmental adaptability in the rhizosphere. The presence of *xerC* recombinase further supports genetic flexibility. Notably, multiple conjugation-related genes (*traD, traC, traN, traV*) along with the antirestriction gene *klcA* indicate that the plasmid is likely conjugative, facilitating horizontal transfer of advantageous traits.

### Prediction and characterization of secondary metabolite biosynthetic gene clusters (BGCs)

**Fig 5:**
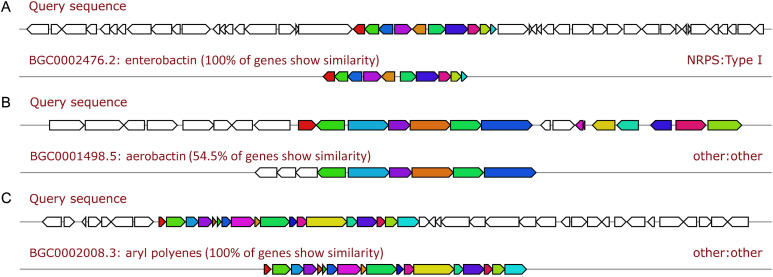
Secondary metabolite biosynthetic gene clusters (BGCs) in *E. roggenkampii* OSNO4 genome identified using antiSMASH. **(A)** Enterobactin, **(B)** aerobactin and **(C)** aryl polyenes.

Secondary metabolite biosynthetic gene clusters (BGCs) analysis using AntiSMASH identified a total of six clusters in the *E. roggenkampii* OSNO4 genome, comprising terpene-precursor, non-ribosomal peptide-metallophore (NRP-metallophore), nonribosomal peptide-independent siderophore (NI-siderophore), azole-containing ribosomally synthesized and post-translationally modified peptide (azole-containing RiPP), homoserine lactone, and aryl polyene types. Among these, the enterobactin, aerobactin, and aryl polyene clusters exhibited amino acid sequence homology of 100%, 54.5%, and 100%, respectively, with previously characterized BGCs. The remaining clusters showed no significant similarity to known gene clusters, indicating the potential presence of novel biosynthetic pathways (Fig 5, S7 Data). The enterobactin cluster harbors key biosynthetic genes, including *entF, entC,* and *entA,* while the aerobactin cluster contains *iucA* and *iucC*. In the aryl polyene cluster, *fabF_1* was identified as a core biosynthetic gene. Overall, these findings highlight both conserved and potentially novel secondary metabolite biosynthetic capacities in *E. roggenkampii* OSNO4, underscoring its prospective functional and ecological significance.

### Genomic screening of antibiotic resistance and virulence-associated genes

Genomic analysis using CARD identified several antibiotic resistance-related genes in *E. roggenkampii* OSNO4, predominantly associated with multidrug efflux systems and regulatory networks (S8 Data). Major efflux components, including *acrAB-tolC, oqxAB, mdtBC, and emrB,* were identified, indicating a strong capacity for active extrusion of a wide range of antimicrobial compounds. The presence of regulatory genes (*marA, ramA, baeR, cpxA, H-NS*) indicates coordinated control under environmental stress conditions, while *FosA2* and *MIR-9* (β-lactamase) represent specific enzymatic resistance mechanisms.

VFDB analysis revealed a limited set of virulence-associated genes, including *csgG, entB,* and *ompA* (S9 Data). These genes are implicated in biofilm formation, siderophore-mediated iron acquisition, and environmental adaptability, respectively, highlighting their roles in rhizosphere colonization and interaction.

### Pan-genome analysis

**Fig 6:**
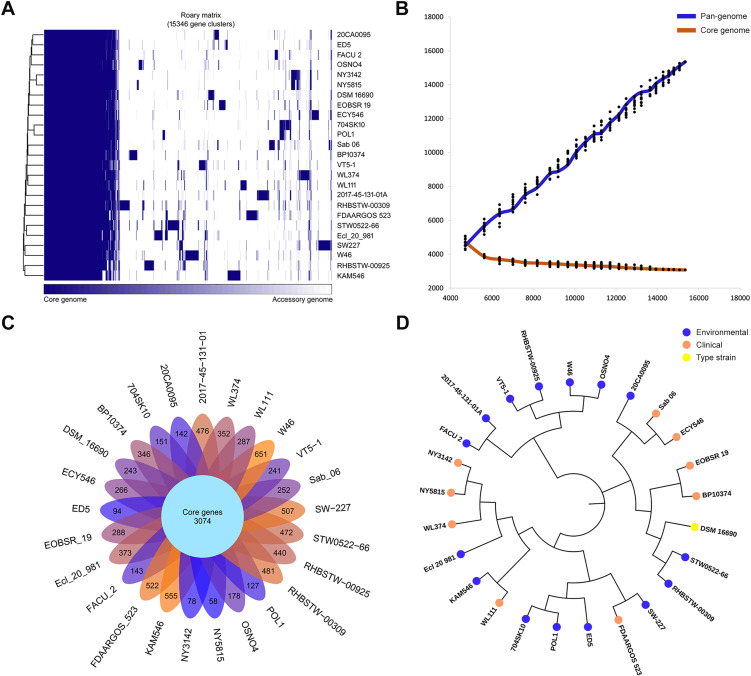
Pan-genome analysis of *E. roggenkampii* strains. **(A)** Gene presence-absence matrix illustrating pan-genome-based clustering across the analyzed strains, **(B)** pan-genome accumulation curve depicting the expansion of the total gene repertoire with the addition of genomes, **(C)** flower plot showing the distribution of strain-specific (unique) genes, **(D)** phylogenetic tree constructed based on the core genome alignment.

The pan-genome analysis using 25 *E. roggenkampii* (S10 Data) including OSNO4 revealed total 15,346 genes, categorized into 3,074 (20.03%) core genes, 377 (2.46%) soft core genes, 1,647 (10.73%) shell genes and 10,248 (66.78%) cloud genes (Fig 6A, S11 Data). Gene accumulation curves showed a decrease in core genome (orange line) size and a simultaneous expansion of the pan-genome (blue line) with the addition of more genome sequences (Fig 6B), indicating an “open” pan-genome consistent with Heaps’ law [77], as further supported by an α value of 0.11. The number of strain-specific genes differed among the isolates, spanning from 58 to 651 genes per strain, which reflects substantial genetic diversity (Fig 6C). A phylogenetic tree based on the core genome of these strains further showed that OSNO4 grouped within a clade of environmental isolates, including W46, RHBSTW-00925, VT5-1, 2017-45-131-01A, and FACU 2 (blue dots) (Fig 6D). While clinical strains (orange dots) were dispersed among multiple distinct clades within the tree.

### Effect of *E. roggenkampii* OSNO4 inoculation on rice growth under salt stress

**Fig 7:**
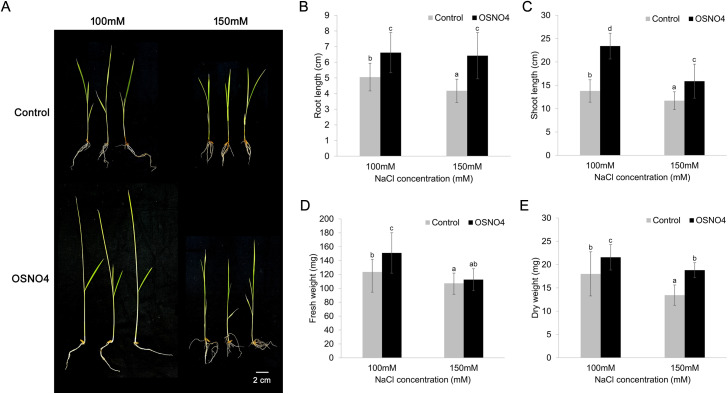
Effect of *E. roggenkampii* OSNO4 inoculation on rice (*Oryza sativa* L.) seedlings under salinity stress. **(A)** Seedlings growth under 100 and 150 mM NaCl, **(B-E)** bar charts showing changes in root length **(B)**, shoot length **(C)**, fresh weight **(D)**, and dry weight **(E)** in OSNO4 inoculated plants compared with non-inoculated controls.

The application of *E. roggenkampii* OSNO4 significantly improved both germination and early growth of rice seedlings under salt stress conditions provided in the laboratory (Fig 7, S12 Data). At 100 mM NaCl, germination increased from 52.22% to 85.56%, along with notable enhancements in root length (5.05 cm vs. 6.62 cm), shoot length (13.79 cm vs. 23.39 cm), fresh weight (123.68 mg vs. 150.96 mg), and dry weight (18.00 mg vs. 21.56 mg). Similarly, under 150 mM NaCl stress, inoculation raised germination from 42.22% to 72.22%, with corresponding increases in root length (4.18 cm vs. 6.42 cm), shoot length (11.70 cm vs. 15.89 cm), fresh weight (107.28 mg vs. 112.56 mg), and dry weight (13.44 mg vs. 18.80 mg).

### Antibiotic susceptibility and hemolysis profile of *E. roggenkampii* OSNO4

**Fig 8:**
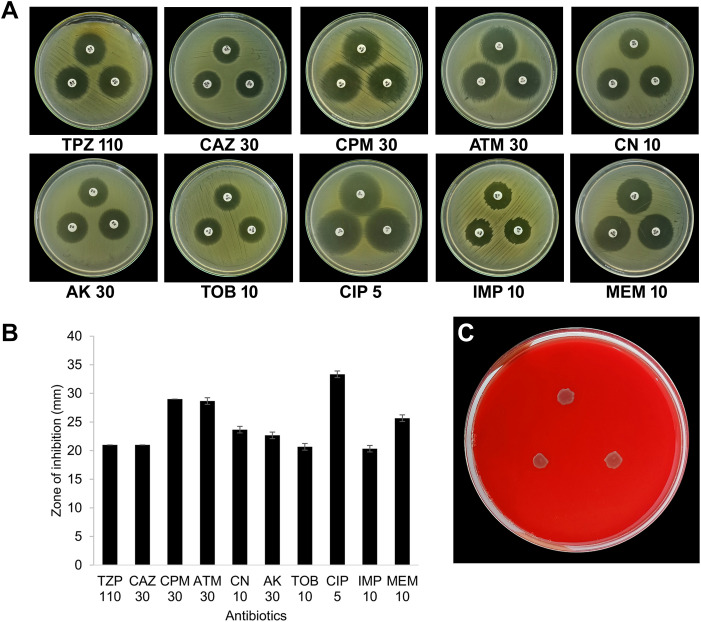
Antibiotic susceptibility profiling and hemolysis assay of *E. roggenkampii* OSNO4. **(A)** Disc diffusion assay for antibiotic sensitivity, **(B)** measurement of inhibition zone diameters, **(C)** hemolysis test on blood agar medium.

*E. roggenkampii* OSNO4 demonstrated broad-spectrum susceptibility pattern against all tested antibiotics (Fig 8A, Fig 8B). The isolate showed the highest zone of inhibition against ciprofloxacin (33.33 mm), followed by cefepime (29.00 mm) and aztreonam (28.67 mm). Intermediate inhibitory effects were observed with meropenem (25.67 mm), gentamicin (23.67 mm), and amikacin (22.67 mm), whereas slightly lower inhibition zones were recorded for piperacillin-tazobactam (21.00 mm), ceftazidime (21.00 mm), tobramycin (20.67 mm), and imipenem (20.33 mm). No hemolytic activity was detected on blood agar, as evidenced by the absence of any clear zones surrounding the colonies, indicating γ-hemolysis (Fig 8C).

## Discussion

The use of beneficial microorganisms in agriculture is becoming increasingly important for enhancing crop productivity and mitigating abiotic stresses in the context of climate variability [78,79]. In this context, genome-assisted characterization of plant growth-promoting rhizobacteria (PGPR) has emerged as a valuable approach for assessing their functional potential and ecological adaptability for sustainable agricultural applications [80,81]. The present study provides an integrated phenotypic and genomic characterization of the halotolerant rice rhizosphere isolate *Enterobacter roggenkampii* OSNO4. The results reveal its genomic basis of multifunctional plant growth-promoting potential, evidenced by phenotypic assays that confirm its functional reliability, highlighting that effective PGPR must harbor and express relevant genes *in situ* [82,83].

The genomic architecture of *E. roggenkampii* OSNO4 exemplifies its advanced metabolic capacity for plant growth-promotion through highly coordinated nutrient-solubilization pathways, consistent with findings reported for other *Enterobacter* species [84,85]. The presence of overlapping high- and low-affinity transport networks, modulated by dedicated regulatory cascades, enables the strain to maintain nutritional homeostasis across steep concentration gradients in fluctuating soil environments [86,87]. Crucially, the metabolic integration of central carbon pathways and pyrroloquinoline quinone (*pqq*) biosynthesis serves as the principle biochemical driver for inorganic mineral solubilization, fueled by the production of organic acids [88]. This metabolic flexibility, coupled with robust, redundant potassium scavenging systems, provides the necessary bioenergetic foundation for OSNO4 to significantly enhance nutrient bioavailability in the rhizosphere.

Similarly, the detected IAA production (38.34 µg/ml) is consistent with the identification of tryptophan-dependent biosynthetic pathways, suggesting its involvement in regulating root development and overall plant growth regulation [89,90]. The detection of the indole-3-pyruvate (IPA) pathway, marked by the presence of key aminotransferase and decarboxylase genes, denotes a highly calibrated auxin production system [91]. Unlike certain high-IAA-producing clinical isolates that can suppress root elongation by overstimulating ethylene biosynthesis through the ACC-mediated pathway [91,92], OSNO4 appears to maintain a moderate, physiologically optimal IAA synthesis. Such fine-tuned hormonal regulation promotes lateral root proliferation and root hair density, thereby expanding the total absorptive surface area of the host plant, a critical survival mechanism under nutrient-limited or saline conditions [93]. The integration of diverse transport systems, regulatory networks, and biosynthetic pathway indicates a tightly coordinated system regulating nitrogen and iron homeostasis in OSNO4, which may collectively contribute to its PGP functions [94,95]. Its versatile nitrogen machinery allows the strain to adapt to fluctuating soil conditions by switching between energy-efficient ammonium assimilation and alternative nitrate, nitrite, or organic urea utilizing pathways to meet metabolic requirements [96]. Simultaneously, the presence of high affinity catecholate-type siderophore systems facilitates efficient iron sequestration, thereby restricting ferric iron availability to competing rhizospheric phytopathogens while maintaining cellular iron homeostasis [97].

The observed alleviation of salinity stress in rice seedlings by *E. roggenkampii* OSNO4 underscores the significant role of PGPR equipped with robust osmoregulatory systems [98,99]. Under saline conditions, plants experience osmotic imbalance and ion toxicity [100]; however, the genomic architecture of OSNO4 directly addresses these challenges. The presence of glycine betaine biosynthesis (*betAB*) and transport machinery (*proVWX*, *proP*), together with pathways involved in the synthesis of compatible solutes such as trehalose (*otsAB*) and proline (*proABC*), allows the isolate to maintain its internal turgor pressure without accumulating toxic levels of inorganic ions [101,102]. In parallel, mechanosensitive channels (*mscL*, *mscS*) and specialized ion transport systems such as potassium uptake operons (*kdpABCDE*) and Na^+^/H^+^ antiporters (*nhaA*, *nhaB*) facilitate intracellular ionic homeostasis [103,104]. These mechanisms, coordinated through regulatory networks such as *envZ*/*ompR* and *phoPQ*, along with general stress-response proteins including *osmY* and *dps*, support bacterial survival and high metabolic activity within the rhizosphere under saline stress [105–107]. As a result, the sustained bacterial population may establish a protective microenvironment around the host root system, thereby lowering localized influx of Na^+^ and enhancing moisture and nutrient accessibility [108]. This bacterial-mediated homeostatic buffering likely mitigates the physiological constraints on the host, translating into the preservation of cell elongation and metabolic activity in the emerging rice seedlings exposed to severe salinity stress.

The biocontrol potential of PGPR is generally associated with their ability to suppress phytopathogens through multiple coordinated mechanisms [109,110]. Here, the antagonistic effect of strain OSNO4 against *Fusarium concentricum* is substantiated by both phenotypic inhibition assays and genomic features. The detection of genes encoding chitin-degrading enzymes and carbohydrate-active proteins suggests a direct mechanism of fungal cell wall degradation [111,112]. Whereas the presence of both catecholate (enterobactin) and hydroxamate (aerobactin) siderophore systems gives OSNO4 a distinct competitive advantage over *F. concentricum* by effectively starving the fungal pathogen of essential Fe^3+^ ions [15,110,113]. Moreover, the detection of aryl polyene and RiPP-related clusters, along with several uncharacterized BGCs, points to the potential production of novel bioactive compounds, which could contribute to its multifaceted biocontrol mechanisms [114]. The identification of these uncharacterized BGCs underscores a substantial repository of secondary metabolites with potential importance for sustained soil suppressiveness and the discovery of novel compounds [115].

Comparative pan-genome analysis of *E. roggenkampii*, including strain OSNO4, demonstrated a limited set of conserved core genes alongside a substantial proportion of accessory and strain-specific genes, highlighting pronounced genomic plasticity within the species. The observed expansion of the pan-genome size with the inclusion of additional genomes, together with a low α value (0.11), indicates an open pan-genome architecture and suggests ongoing gene acquisition and adaptive evolution [77]. Such genomic diversity is reflected in the variation of strain-specific genes and likely contributes to adaptation in complex soil environments [116]. Notably the clustering of OSNO4 within a lineage composed predominantly of environmental isolates, rather than clinical strains, indicates distinct genomic streamlining [117,118]. In rhizospheric habitats, such evolutionary divergence typically drives the acquisition of fitness-conferring genes such as those involved in nutrient mobilization (*pst*, *nif*, or *sul* clusters) and motility, while facilitating the adaptive pseudogenization or deletion of major virulence determinants (*inv* or *hly* homologs) that are otherwise maintained by clinical strains under host-selection pressure [119]. This ecological specialization may additionally be facilitated by the strain’s mobile plasmid, which functions as a specialized genetic vehicle for survival in the rhizosphere [120]. Through the combination of efficient conjugative transfer systems and molecular defense strategies that circumvent host restriction barriers, the plasmid may promote the stable maintenance and horizontal dissemination of stress tolerance and survival-critical stress response traits across the soil microbiome [121,122].

However, when considering PGPR for agricultural application, evaluation of biosafety becomes a key requirement, particularly for members of the genus *Enterobacter*, which include many clinically relevant species [123–125]. Despite possessing multiple resistance-associated genes (e.g., *acrAB*-*tolC*, *oqxAB*, *fosA2*, and *MIR-9*), the broad-spectrum phenotypic susceptibility displayed by *E. roggenkampii* OSNO4 highlights a common discrepancy in environmental isolates where silent genomic resistance mechanisms are unexpressed under standard *in vitro* conditions. The activity of these efflux systems and specific enzymatic determinants is likely tightly controlled by networks like *marA* and *ramA*, remaining dormant until triggered by specific environmental or anthropogenic stress signals [126]. For a PGPR, the predominance of fitness-associated traits over pathogenic determinants further supports its non-pathogenic ecological lifestyle. Genes such as *csgG* associated with biofilm matrix assembly; *entB*, involved in iron scavenging; and *ompA* important for cellular integrity underline a non-pathogenic, adaptive strategy optimized for rhizosphere colonization and symbiotic persistence [127,128]. This ecological specialization toward plant-microbe interaction over virulence is further substantiated by the absolute absence of hemolytic activity [129], supporting OSNO4 as a functionally robust and environmentally safe candidate for biofertilizer applications.

Overall, the plant growth-promoting performance of *E. roggenkampii* OSNO4 is likely driven by the synergistic interaction of multiple functional traits supported by a comprehensive genomic background. Consistency across *in vitro* assays, genomic predictions, and plant responses highlights the reliability of this strain as a climate-resilient bioinoculant, particularly under salinity stress conditions. By demonstrating both agronomic potential and biosafety through an integrated genomic approach, this study satisfies its primary objective of providing a scientifically rigorous alternative to chemical inputs in climate-vulnerable agricultural zones. Further work should focus on field-level validation of OSNO4 across diverse agro-climatic conditions to validate its efficacy and consistency as a bioinoculant. In parallel, analyses of plant biochemical responses together with qRT-PCR-based expression profiling will be essential to map the underlying host-transcriptome responses.

## Conclusion

In conclusion, this study identifies *Enterobacter roggenkampii* OSNO4 as a safe, halotolerant bioinoculant with significant potential for mitigating salinity stress in climate-sensitive agricultural systems. Through the integration of phenotypic characterization and genome-based analyses, we showed that the plant growth-promoting potential of the strain is driven by metabolic traits associated with nutrient mobilization, balanced auxin production, iron acquisition, and osmoregulation under salt stress. Importantly, pan-genome and phylogenomic analyses revealed that OSNO4 clusters within a distinct environmental lineage, where genomic plasticity appears to enhance rhizosphere fitness while reducing the retention of major virulence-associated traits. Together with its broad antibiotic susceptibility profile and complete absence of hemolytic activity, these findings provide evidence that environmentally adapted members of potentially opportunistic genera can undergo niche-specific evolution while maintaining biosafety. Collectively, this work provides a scientifically robust, genome-driven framework for developing microbial alternatives to chemical inputs in salt-affected agricultural ecosystems.

## Supporting information

S1 Data Description of soil samples, bacterial count from each sample and isolated rhizobacteria from rhizosphere of rice. (XLSX)

S2 Data *In vitro* plant growth-promoting features, abiotic stress tolerance and antifungal assay of bacterial strains isolated from the rhizosphere of rice. (XLSX)

S3 Data Biochemical test results of isolate OSNO4. (XLSX)

S4 Data dDDH values of strain OSNO4 with related species. (XLSX)

S5 Data Pairwise average nucleotide identity (ANI) values of OSNO4 with related species. (XLSX)

S6 Data *E. roggenkampii* OSNO4 genes involved in plant growth-promoting activity as predicted by PGPT-Pred tool. (XLSX)

S7 Data Secondary metabolite biosynthetic gene clusters (BGCs) of *E. roggenkampii* OSNO4 identified using AntiSMASH. (XLSX)

S8 Data Genome-wide screening of antibiotic resistant genes in *E. roggenkampii* OSNO4 using CARD database. (XLSX)

S9 Data Genome-wide screening of virulence related genes in *E. roggenkampii* OSNO4 using VFDB database. (XLSX)

S10 Data List of strains used for the pan-genome analysis of *E. roggenkampii* OSNO4. (XLSX)

S11 Data Pan-genome analysis of *E. roggenkampii* OSNO4. (XLSX)

S12 Data Effects of *E. roggenkampii* OSNO4 inoculation on rice (*Oryza sativa* L.) seedling growth under saline conditions (100 mM and 150 mM NaCl).
(XLSX)
